# Anterior-Posterior Instability of the Knee Following ACL Reconstruction with Bone-Patellar Tendon-Bone Ligament in Comparison with Four-Strand Hamstrings Autograft

**DOI:** 10.1155/2013/572083

**Published:** 2013-07-10

**Authors:** A. G. Angoules, K. Balakatounis, E. C. Boutsikari, D. Mastrokalos, P. J. Papagelopoulos

**Affiliations:** ^1^Technological Educational Institute of Athens, Faculty of Health & Care Professions, Athens, Greece; ^2^Egersis, Filoktitis Rehabilitation Center, Athens, Greece; ^3^First Department of Orthopaedics, Athens University School of Medicine, Athens, Greece

## Abstract

*Purpose*. To evaluate anterior-posterior knee laxity using two different autografts. *Material-Methods*. 40 patients, (34 males and 6 women), 17–54 years old (mean: 31), were included in the present study. Group A (4SHS = 20) underwent reconstruction using four-strand hamstrings, and group B (BPBT = 20) underwent reconstruction using bone-patellar tendon-bone autograft. Using the KT-1000 arthrometer, knee instability was calculated in both knees of all patients preoperatively and 3, 6, and 12 months after surgery at the ACL-operated knee. The contralateral healthy knee was used as an internal control group. *Results*. Anterior-posterior instability using the KT1000 Arthrometer was found to be increased after ACL insufficiency. The recorded laxity improved after arthroscopic ACL reconstruction in both groups. However, statistically significant greater values were detected in the bone-patellar tendon-bone group, which revealed reduction of anteroposterior stability values to an extent, where no statistical significance with the normal values even after 3 months after surgery was observed. *Conclusions*. Anterior-Posterior instability of the knee improved significantly after arthroscopic ACL reconstruction. The bone-patellar tendon-bone graft provided an obvious greater stability.

## 1. Introduction

The anterior cruciate ligament (ACL) is a major stabilizing element of the knee since it is the main anatomical structure which prevents the anterior displacement of the tibia relative to the femur [1, 2].

 Simultaneously, it is an important factor for the normal knee movements, since it contributes not only to the static, but also to the dynamic stability of the joint [3].

 Dynamic stability is guaranteed by the presence of specific ligament mechanoreceptors which are considered an essential element for knee proprioception as it has been revealed by a few anatomical and histological studies [3–6]. 

The ACL is the most frequently injured knee ligament especially as regards sports that include movements with sudden direction changes, as knee supports body weight during them [7–9]. These lesions often lead to ligament rupture with subsequent impairment and instability of the knee.

Diagnosis is based presumably on several clinical examinations such as Lachman test, the anterior drawer test, and pivot shift test [2]. This kind of examinations, depending on the extent of the time that is inserted between the examination and the accident, the adeptness, and the experience of the health professional, as well as the body type of the patient, can lead to different results [10]. 

Thus, numerous objectively measurable methods of evaluation have been developed, by using mechanical devices such as KT-1000 which is the most frequently used [10–13].

A significant number of patients with ACL rupture undergo surgical reconstruction that it is carried out with different types of autografts, with bone-patellar tendon-bone ligament and four-strand hamstrings grafts, being the most widely performed, with single and double bundle techniques [14–17]. 

In this paper, the anterior-posterior instability of the knee after ACL rupture and deficiency, as well as joint's restoration stability after ACL reconstruction with the aforementioned autografts, was studied, using the KT-1000 arthrometer.

## 2. Materials and Methods

Forty nonprofessionals athletes, with clinically and MRI recognizable unilateral ACL rupture and knee insufficiency, underwent ACL reconstruction with two different types of autografts. There were 34 men and 6 women, with a mean age of 31 years (range 17–54 years). Exclusion criteria were ages smaller than 16 years, postoperative complications, presence of injuries or surgery, pain or function wastage in the corresponding knee within the last 6 months, inability of cooperation, and psychiatric diseases, as well as alcoholism or usage of addictive substances.

The subjects were randomized into 2 groups according to their gender and age. Group A included 20 patients (16 men and 4 women) with ACL rupture that underwent reconstruction using four-strand autografts (4SHS). Group B was consisted of 20 patients (18 men and 2 women) that underwent reconstruction with bone-patellar-tendon-bone (BPTB) ligament.

**Figure 1:**
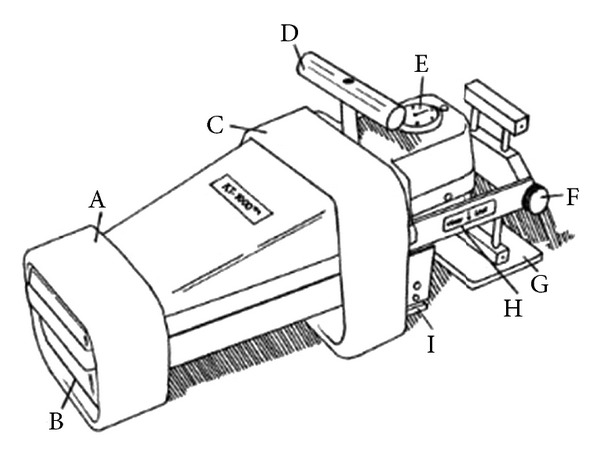


All patients that participated in the undersigned study were operated arthroscopically with the same technique, while they followed the exact same physical rehabilitation program. The objective measurable anteroposterior knee instability was calculated using a KT-1000 arthrometer (MEDmetric, Corporation, San Diego, CA, USA) [18] (Figure 1) preoperatively and postoperatively, in the 3rd, the 6th, and 12th months after ACL reconstruction.

The knees of the patient were placed in 30 degrees of flexion, with the heel symmetrically placed on a foot rest, so that the tibiae could both stand in 15 degrees of external rotation. In the knee, there were exerted sequentially forces equal to 67 N, 89 N with anterior direction, and the tibial displacement was counted in millimeters between 89 N and 67 N [19, 20].

The study has been approved by the Institutional Review Board/Ethics Committee of the authors' institutions.

## 3. Statistical Analysis

The evaluation variables were described using the number of participants (*N*), the mean values or medians, if it was estimated that there was not a normal distribution of values and standard deviations. 

In order to control the interaction between the surgical technique factor and the time factor, the mixed model of variance analysis with 2 factors was used (two way ANOVA mixed model). 

For the longitudinal comparison of variables per group (baseline versus values of 3rd month versus values of 6th month versus values of 12th month), the model of variance analysis with one factor repeated measures was used (one factor repeated measures ANOVA).

For the analysis of the differences between group A and group B over time, the percentage of changes in comparison with the baseline group was estimated, for the period of 3, 6, and 12 months. 

The comparison of percentage changes from baseline between the two groups was performed with the *t*-test for independent samples (independent samples *t*-test). The comparison of percentage changes from baseline variables between the healthy and the injured knee was performed using the *t*-test for paired samples (paired sample *t*-test).

All tests were two sided with significance level, *P* = 0.05. Statistical analyses were done using the SPSS vr 13.00 (Statistical Package for the Social Sciences, SPSS Inc., Chicago, IL, USA).

## 4. Results

In group A, as well as in group B, a statistically considerable variation amongst the normal and the injured knee was recorded preoperatively (Table 1). 

Secondarily, it followed the control of the KT-1000 variable overtime separately for every type, using the one factor repeated measures ANOVA.

Statistically significant variation was recorded amongst the absolute changes of variable KT-1000 in Group A (*P* < 0.0005). Based on the performed pairwise comparisons, variation from the preoperative values it was recorded (Table 2).

Statistically conspicuous change was also recorded amongst the absolute changes of variable KT-1000 in Group B (Table 3).

 The pairwise comparisons showed differentiation between all measurements and the preoperative values. The percentage change of KT-1000 from baseline to 12 months was also assayed, using the parametric *t*-test for independent samples, and the nonparametric Mann-Whitney test, while the results were expressed as median when normal distribution was violated.

A statistically significant alteration amongst the two types of percentage changes from baseline to 3, 6, and 12 months, respectively, for the variable KT-1000 was calculated (Tables 4 and 5).

## 5. Discussion

In this paper, the anterior laxity of the knee after rupture and insufficiency of the ACL was studied, as well as the variation of this parameter after ligament reconstruction with BPBT in comparison with 4SHS graft.

The evaluation of knee instability was performed with KT 1000 arthrometer.

This medical device is a useful instrument that performs objectively the relative movement of the tibia over the femur after ACL reconstruction [11, 12, 19]. 

The reliability of this method is recorded between 0,83 and 0,88, whilst the sensitivity is calculated up to 90%. Thus, it is proposed that it is capable of replacing the Lachman-Noulis test [13].

The anterior-posterior instability of the knee after rupture and insufficiency of the ACL, as measured by the KT-1000, reveals increased values in this study which is in line with the international literature [12, 19, 21].

This instability shows improvement after reconstruction of the deficient ligament. Both grafts are used to improve the laxity of the knee, with the BPBT being the most appropriate graft for the desirable result.

In fact, the group that underwent the operation with the specific type of graft displays a decrease of the counted instability in a degree that there are no differences of statistical importance as regards the preoperative values of the injured knee during the 6th postoperative month. In previous studies, it is also recorded a higher percentage of patients with a difference less than 3 mm in anteroposterior laxity amongst both knees and a generally better stability in the BPBT group in comparison with the 4SHS group [22–31]. Fewer studies could not detect differences in the knee stability amongst the two grafts with this method [24, 31–35] marginally improved or they recorded anteroposterior instability, but not in a statistically important level in the hamstrings group [36, 37]. Holm et al. in a recent research after a long-term observation, and after ligament reconstruction, report similar results in the restoration of the anteroposterior instability with both grafts [38].

Both autografts have sufficient tensile strength and provide adequate stability to the knee [16, 39, 40].

As the patellar tendon graft has been associated with donor-site morbidity such as anterior knee pain, loss of sensation, patellar fracture, inferior patellar contracture, and loss of extension torque, hamstrings use as an alternative graft option has gained an increased popularity in the last years [34, 41]. The latter is thought to be followed by fewer complications [17, 42, 43].

 Different surgical procedures such as the transtibial and the arthroscopic anteromedial portal technique have been used for the drilling of the femoral tunnel in ACL reconstruction using 4SHS with comparable results on the most evaluated parameters [44]. As regards the fixation techniques, it is accepted that cross-pin femoral devices provide a high fixation strength and sufficient resistance against slippage in comparison with the conventional interference screws [45].

Different parameters are considered to influence the final functional result following ACL reconstruction, apart from the anteroposterior stability established [46]. ACL is both a static and a dynamic element of vital importance for the functionality of the knee [3, 47]. These particularly complex sensorimotor mechanisms exist within a secure and steady environment which is the result of the static mechanical improvement provided by the graft itself [5]. 

Obviously, apart from the graft choice, the postoperative rehabilitation is capable of improving the end result of the operation, since it contributes to the restoration of proprioceptive deficits after rupture and impairment of the ACL [47]. Predominantly, patients with significant proprioceptive insufficiency may be helped by participating in physical therapy programs that focus on proprioception of the lower limb and by returning to functional activities, in addition to standard rehabilitation programs that focus mainly on the restoration of muscle strength [48, 49].

## 6. Conclusion

After ACL rupture and deficiency, increased values of anterior-posterior knee instability are recorded and accurately measured with KT-1000 arthrometer.

This instability can be restored after 6 months following ACL reconstruction with bone-patellar-bone-tendon or four-strand hamstrings autografts. Although both grafts are capable of restoring the anterior laxity of the joint, the BPBT graft appears to excel, as it ensures greater stability. 

Additional clinical trials are required to indicate the ideal selection of the graft for every individual that suffered from ACL rupture and insufficiency, apart from the static and the notably dynamic role of the ligament.

In particularly for patients undergoing ACL reconstruction using 4SHS, programmes for neuromuscular control and proprioception enhancement should be necessarily planned.

## Figures and Tables

**Table 1:** 

KT1000		*N*	Mean value	Standard deviation	*P* value
Group A	ACL-deficient	20	6.7000	1.94936	**<0.0005**
	Healthy	20	2.0000	1.21395	

Group B	ACL-deficient	20	6.2500	1.77334	**<0.0005**
	Healthy	20	1.6500	.98809	

**Table 2:** 

Time of evaluation (months)	Group A						
	*N*	Mean	SD	Pairwise comparisons			
				Baseline	3 months	6 months	12 months
Baseline	20	6.7000	1.94	—	*P* < 0.0005	*P* < 0.0005	*P* < 0.0005
3	20	3.1000	1.29	—	—	N.S	N.S
6	20	3.0000	1.29	—	—	—	N.S
12	20	2.8000	1.28	—	—	—	—
Overall Sig.	***P* < 0.0005**						

SD: standard deviation; overall sig: overall significance.

**Table 3:** 

Time of evaluation (months)	Group B						
	*N*	Mean	SD	Pairwise comparisons			
				Baseline	3 months	6 months	12 months
Baseline	20	6.2500	1.77	—	*P* < 0.0005	*P* < 0.0005	*P* < 0.0005
3	20	1.9500	1.39	—	—	N.S	N.S
6	20	1.7500	1.48	—	—	—	N.S
12	20	1.7500	1.48	—	—	—	—
Overall sig.	***P* < 0.0005**						

SD: standard deviation; overall sig: overall significance.

**Table 4:** 

Median % change from baseline		*N*	Mean	Mean standard error	*P* value
3 months	Graft A	20	−53.9518	4.42350	**0.025**
	Graft B	20	−68.9266	4.65002	

6 months	Graft A	20	−55.2217	4.54732	**0.009**
	Graft B	20	−73.2599	4.78314	

12 months	Graft A	20	−58.3050	4.54709	**0.029**
	Graft B	20	−73.2599	4.78314	

**Table 5:** 

Median % change from baseline		*N*	Median	*P* value
3 months	Graft A	20	−52.78	0.033
	Graft B	20	−66.67	

6 months	Graft A	20	−53.57	0.013
	Graft B	20	−77.50	

12 months	Graft A	20	−58.57	0.052
	Graft B	20	−77.50	

## References

[1] Fu FH, Harner CD, Johnson DL, Miller MD, Woo SL (1994). Biomechanics of knee ligaments: basic concepts and clinical application. *Instructional course lectures*.

[2] Woo SL-Y, Debski RE, Withrow JD, Janaushek MA (1999). Biomechanics of knee ligaments. *American Journal of Sports Medicine*.

[3] Johansson H, Sjolander P, Sojka P (1991). A sensory role for the cruciate ligaments. *Clinical Orthopaedics and Related Research*.

[4] Hogervorst T, Brand RA (1998). Mechanoreceptors in joint function. *Journal of Bone and Joint Surgery*.

[5] Angoules AG, Mavrogenis AF, Dimitriou R (2011). Knee proprioception following ACL reconstruction; a prospective trial comparing hamstrings with bone-patellar tendon-bone autograft. *The Knee*.

[6] Dhillon MS, Bali K, Prabhakar S (2011). Proprioception in anterior cruciate ligament deficient knees and its relevance in anterior cruciate ligament reconstruction. *Indian Journal of Orthopaedics*.

[7] Bollen SR, Scott BW (1996). Rupture of the anterior cruciate ligament—a quiet epidemic?. *Injury*.

[8] Johnson RJ (1982). The anterior cruciate: a dilemma in sports medicine. *International Journal of Sports Medicine*.

[9] Miyasaka K, Daniel D, Stone M (1991). The incidence of knee ligament injuries in general population. *The American Journal of Knee Surgery*.

[10] Collette M, Courville J, Forton M, Gagnière B (2012). Objective evaluation of anterior knee laxity; comparison of the KT-1000 and GNRB arthrometers. *Knee Surgery, Sports Traumatology, Arthroscopy*.

[11] Arneja S, Leith J (2009). Review article: validity of the KT-1000 knee ligament arthrometer. *Journal of Orthopaedic Surgery*.

[12] Daniel DM, Malcom LL, Losse G (1985). Instrumented measurement of anterior laxity of the knee. *Journal of Bone and Joint Surgery A*.

[13] Neeb TB, Aufdemkampe G, Wagener JHD, Mastenbroek L (1997). Assessing anterior cruciate ligament injuries: the association and differential value of questionnaires, clinical tests, and functional tests. *Journal of Orthopaedic and Sports Physical Therapy*.

[14] Biau DJ, Tournoux C, Katsahian S, Schranz P, Nizard R (2007). ACL reconstruction: a meta-analysis of functional scores. *Clinical Orthopaedics and Related Research*.

[15] Xu Y, Ao YF, Wang JQ, Cui G-Q (2013). Prospective randomized comparison of anatomic single- and double-bundle anterior cruciate ligament reconstruction. *Knee Surgery, Sports Traumatology, Arthroscopy*.

[16] Forster MC, Forster IW (2005). Patellar tendon or four-strand hamstring? A systematic review of autografts for anterior cruciate ligament reconstruction. *The Knee*.

[17] Li S, Chen Y, Lin ZW, Cui W, Zhao J, Su W (2012). A systematic review of randomized controlled clinical trials comparing hamstring autografts versus bone-patellar tendon-bone autografts for the reconstruction of the anterior cruciate ligament. *Archives of Orthopaedic and Trauma Surgery*.

[18] MEDrnetric Corporation *KT-1000/KT-2000 Knee Ligament Arthrometer User's Guide*.

[19] Daniel DM, Stone ML, Sachs R, Malcom L (1985). Instrumented measurement of anterior knee laxity in patients with acute anterior cruciate ligament disruption. *American Journal of Sports Medicine*.

[20] Kowalk DL, Wojtys EM, Disher J, Loubert P (1993). Quantitative analysis of the measuring capabilities of the KT-1000 knee ligament arthrometer. *American Journal of Sports Medicine*.

[21] Bach BR, Warren RF, Flynn WM, Kroll M, Wickiewiecz TL (1990). Arthrometric evaluation of knees that have a torn anterior cruciate ligament. *Journal of Bone and Joint Surgery A*.

[22] Ejerhed L, Kartus J, Sernert N, Köhier K, Karlsson J (2003). Patellar tendon or semitendinosus tendon autografts for anterior cruciate ligament reconstruction? A prospective randomized study with a two-year follow-up. *American Journal of Sports Medicine*.

[23] Freedman KB, D’Amato MJ, Nedeff DD, Kaz A, Bach BR (2003). Arthroscopic anterior cruciate ligament reconstruction: a metaanalysis comparing patellar tendon and hamstring tendon autografts. *American Journal of Sports Medicine*.

[24] Marder RA, Raskind JR, Carroll M (1991). Prospective evaluation of arthroscopically assisted anterior cruciate ligament reconstruction. Patellar tendon versus semitendinosus and gracilis tendons. *American Journal of Sports Medicine*.

[25] Engebretsen L, Benum P, Fasting O, Molster A, Strand T (1990). A prospective, randomized study of three surgical techniques for treatment of acute ruptures of the anterior cruciate ligament. *American Journal of Sports Medicine*.

[26] Corry IS, Webb JM, Clingeleffer AJ, Pinczewski LA (1999). Arthroscopic reconstruction of the anterior cruciate ligament. A comparison of patellar tendon autograft and four-strand hamstring tendon autograft. *American Journal of Sports Medicine*.

[27] Otero AL, Hutcheson LA (1993). A comparison of the doubled semitendinosus/gracilis and central third of the patellar tendon autografts in arthroscopic anterior cruciate ligament reconstruction. *Arthroscopy*.

[28] Yunes M, Richmond JC, Engels EA, Pincweski LA (2001). Patellar versus hamstring tendons in anterior cruciate ligament reconstruction: a meta-analysis. *Arthroscopy*.

[29] Feller JA, Webster KE, Gavin B (2001). Early post-operative morbidity following anterior cruciate ligament reconstruction: patellar tendon versus hamstring graft. *Knee Surgery, Sports Traumatology, Arthroscopy*.

[30] Anderson AF, Snyder RB, Lipscomb AB (2001). Anterior cruciate ligament reconstruction. A prospective randomized study of three surgical methods. *American Journal of Sports Medicine*.

[31] Shaieb MD, Kan DM, Chang SK, Marumoto JM, Richardson AB (2002). A prospective randomized comparison of patellar tendon versus semitendinosus and gracilis tendon autografts for anterior cruciate ligament reconstruction. *American Journal of Sports Medicine*.

[32] Matsumoto A, Yoshiya S, Muratsu H (2006). A comparison of bone-patellar tendon-bone and bone-hamstring tendon-bone autografts for anterior cruciate ligament reconstruction. *American Journal of Sports Medicine*.

[33] Beard DJ, Anderson JL, Davies S, Price AJ, Dodd CAF (2001). Hamstrings versus patella tendon for anterior cruciate ligament reconstruction: a randomised controlled trial. *The Knee*.

[34] Aune AK, Holm I, Risberg MA, Jensen HK, Steen H (2001). Four-strand hamstring tendon autograft compared with patellar tendon-bone autograft for anterior cruciate ligament reconstruction: a randomized study with two-year follow-up. *American Journal of Sports Medicine*.

[35] Lidén M, Ejerhed L, Sernert N, Laxdal G, Kartus J (2007). Patellar tendon or semitendinosus tendon autografts for anterior cruciate ligament reconstruction: a prospective, randomized study with a 7-year follow-up. *American Journal of Sports Medicine*.

[36] Webster KE, Feller JA, Hameister KA (2001). Bone tunnel enlargement following anterior cruciate ligament reconstruction: a randomised comparison of hamstring and patellar tendon grafts with 2-year follow-up. *Knee Surgery, Sports Traumatology, Arthroscopy*.

[37] Wagner M, Kääb MJ, Schallock J, Haas NP, Weiler A (2005). Hamstring tendon versus patellar tendon anterior cruciate ligament reconstruction using biodegradable interference fit fixation: a prospective matched-group analysis. *American Journal of Sports Medicine*.

[38] Holm I, Oiestad BE, Risberg MA, Aune AK (2010). No difference in knee function or prevalence of osteoarthritis after reconstruction of the anterior cruciate ligament with 4-strand hamstring autograft versus patellar tendon-bone autograft: a randomized study with 10-year follow-up. *The American Journal of Sports Medicine*.

[39] Herrington L, Wrapson C, Matthews M, Matthews H (2005). Anterior Cruciate Ligament reconstruction, hamstring versus bone-patella tendon-bone grafts: a systematic literature review of outcome from surgery. *The Knee*.

[40] Noyes FR, Butler DL, Grood ES (1984). Biomechanical analysis of human ligament grafts used in knee-ligament repairs and reconstructions. *Journal of Bone and Joint Surgery A*.

[41] Kartus J, Magnusson L, Stener S, Brandsson S, Eriksson BI, Karlsson J (1999). Complications following arthroscopic anterior cruciate ligament reconstruction: a 2-5-year follow-up of 604 patients with special emphasis on anterior knee pain. *Knee Surgery, Sports Traumatology, Arthroscopy*.

[42] Biau DJ, Tournoux C, Katsahian S, Schranz PJ, Nizard RS (2006). Bone-patellar tendon-bone autografts versus hamstring autografts for reconstruction of anterior cruciate ligament: meta-analysis. *British Medical Journal*.

[43] Li S, Su W, Zhao J (2011). A meta-analysis of hamstring autografts versus bone-patellar tendon-bone autografts for reconstruction of the anterior cruciate ligament. *The Knee*.

[44] Koutras G, Papadopoulos P, Terzidis IP, Gigis I, Pappas E (2012). Short-term functional and clinical outcomes after ACL reconstruction with hamstrings autograft: transtibial versus anteromedial portal technique. *Knee Surgery, Sports Traumatology, Arthroscopy*.

[45] Hantes ME, Dailiana Z, Zachos VC, Varitimidis SE (2006). Anterior cruciate ligament reconstruction using the Bio-TransFix femoral fixation device and anteromedial portal technique. *Knee Surgery, Sports Traumatology, Arthroscopy*.

[46] Ageberg E (2002). Consequences of a ligament injury on neuromuscular function and relevance to rehabilitation—using the anterior cruciate ligament-injured knee as model. *Journal of Electromyography and Kinesiology*.

[47] Hewett TE, Paterno MV, Myer GD (2002). Strategies for enhancing proprioception and neuromuscular control of the knee. *Clinical Orthopaedics and Related Research*.

[48] Boca IC, Dan M (2013). The effectiveness of proprioceptive neuromuscular facilitation techniques and hidrotherapy to improve knee stability after anterior cruciate ligament reconstruction. *British Journal of Sports Medicine*.

[49] Risberg MA, Holm I, Myklebust G, Engebretsen L (2007). Neuromuscular training versus strength training during first 6 months after anterior cruciate ligament reconstruction: a randomized clinical trial. *Physical Therapy*.

